# Personalised Nutritional Recommendations Based on Individual Post-Prandial Glycaemic Responses Improve Glycaemic Metrics and PROMs in Patients with Type 2 Diabetes: A Real-World Assessment

**DOI:** 10.3390/nu14102123

**Published:** 2022-05-19

**Authors:** Madlen Ungersboeck, Xiaowen Tang, Vanessa Neeff, Dominic Steele, Pascal Grimm, Matthew Fenech

**Affiliations:** Una Health GmbH, Rosenthaler Str. 13, 10119 Berlin, Germany; madlen@unahealth.io (M.U.); xiaowen@unahealth.io (X.T.); vanessa@unahealth.io (V.N.); dominic@unahealth.io (D.S.); pascal@unahealth.io (P.G.)

**Keywords:** personalised nutrition, type 2 diabetes, continuous glucose monitoring, digital tools

## Abstract

The recommended first-line therapy in type 2 diabetes (T2D) is lifestyle modification. In many patients, such interventions fail, and disease progresses inexorably to medication requirement. A potential reason for the failure of standard nutritional interventions is the use of generic dietary advice, with no personalisation to account for differences in the effect of food on blood glucose between different individuals. Another is the lack of instant feedback on the impact of dietary modification on glycaemic control, which supports sustained behaviour change. The use of continuous glucose monitoring (CGM) may help address both these shortcomings. We conducted an observational study to explore how personalised nutritional information impacts glycaemic control and patient-reported outcome measures (PROMs) of well-being. Free-living people with T2D eating their normal diet were provided with personalised nutritional recommendations by state-registered nutritionists based on the CGM-enabled analysis of individual post-prandial glycaemic responses (PPGRs). Participants demonstrated considerable inter-individual differences in PPGRs, reductions in post-prandial incremental area under the curve (iAUC) and daytime AUC, and improvements in energy levels, ability to concentrate, and other PROMs. These results suggest a role for personalised nutritional recommendations based on individual-level understanding of PPGRs in the non-pharmaceutical management of T2D.

## 1. Introduction

Type 2 diabetes (T2D) is a serious chronic disease characterised by dysregulated insulin secretion and activity, leading to increased levels of glucose in the bloodstream [1]. In the last few decades, there has been a dramatic increase in the incidence of type 2 diabetes worldwide, with some models predicting 700 million people with diabetes by 2045 [2,3]. Diabetes is the ninth leading cause of mortality globally, largely due to the development of complications including cardiovascular and peripheral vascular disease, retinopathy, neuropathy, and nephropathy. Diabetes and its complications are responsible for high levels of patient morbidity and health expenditure worldwide [1].

Different risk factors for the development of T2D, including genetics, age, and ethnicity, have been described. Lifestyle factors such as diet, physical activity, and stress play a pivotal part in T2D development [4], but interventions designed to improve lifestyle and thereby to slow, halt, or even reverse disease progression have shown limited success [5,6]. One reason for this is that current lifestyle interventions are largely based on generic, one-size-fits-all approaches, and do not take into account individual post-prandial glycaemic responses (PPGRs). A person’s PPGR following a specific meal is largely consistent, but there can be great differences between individuals in this response to the same meal [7,8]. PPGR is also a major contributor to overall glycaemic variability, which is increasingly recognised as an independent predictor of disease progression [7,8,9,10,11,12,13].

Therefore, an understanding of a patient’s specific dietary contributors to dysregulated PPGRs may provide an insight into a worthwhile therapeutic target beyond HbA1c. Secondly, patients find current lifestyle interventions difficult to adhere to, in part due to an inability to rapidly see the positive consequences of dietary change (for example, as reflected in reduced blood sugars); such feedback improves motivation and engagement with these interventions [5,6,9,10].

Technologies that can support the personalised understanding of glycaemic variation and can provide quasi-immediate feedback on the effects of lifestyle changes include continuous glucose monitoring (CGM) and the closely related flash glucose monitoring. Although the benefit for CGM is largely accepted in patients with type 2 diabetes on intensive insulin treatment [14,15,16,17,18], there is increasing evidence that it may play a role in T2D that is not treated with prandial insulin. The use of CGM in this group of patients was associated with improvements in glycaemic control that persisted in the long-term [11], weight loss, and improved diet [12,13]. Indeed, the effects of nutritional interventions on weight, HbA1c, and other lagging indicators of metabolic health and glycaemic control are well-explored, but there is much less understanding about the roles of very relevant and near-real-time indicators of metabolic health and glycaemic control on personalised dietary behaviour [19]. As CGM provides patients and their healthcare professionals with a large amount of data, guidelines have been created to standardise the analysis of PPGRs from CGM data, which rest on the identification of the maximum glucose level achieved after a meal, the difference between this maximum glucose and the value at meal start, and the time taken for the glucose to return to premeal levels [20]. To date, studies analysing the effects of personalised nutrition are generally conducted in highly controlled settings and do not focus on free-living people with their various diets without any intervention by study investigators [7,8]. We are unaware of studies that have systematically assessed the role of CGM in allowing patients and their healthcare providers to identify individual post-prandial glycaemic responses in a real-world setting.

Some state-registered nutritionists can incorporate the results from their clients’ CGM when providing personalised recommendations to them [12]. CGM is available without prescription in Germany, and patients with diabetes (and even people without metabolic disease) self-fund the use of this device. We set out to understand how PPGRs vary between members of a group of free-living patients with type 2 diabetes not on prandial insulin who are eating their normal diet, and to observe the impact of CGM-driven, personalised nutritional recommendations on glycaemic control and on patient-reported outcome measures of diabetes-related well-being. Furthermore, we systematically collected and analysed qualitative data on patient experiences of such a program. This observational study was conducted to understand the value of developing a digital health product along these lines.

## 2. Materials and Methods

### 2.1. Recruitment

The recruitment of potential participants was conducted consecutively via online advertisement on different social media channels between August and October 2021. Potential participants who signed up on the Una Health website were screened in a telephone call to confirm eligibility (Table 1). Forty-two individuals who met all eligibility criteria were selected and were enrolled. At enrolment, participant age, gender, most recent HbA1c value recorded by their medical practitioner, weight, BMI, year of and age at diabetes diagnosis, use of antidiabetic medication, and past medical history were recorded.

### 2.2. Study Design

The study was designed to observe the effect of the provision of dietary modification recommendations based on patients’ post-prandial glycaemic responses to different meals, by five different state-registered nutritionists. All nutritionists had experience of interpreting CGM data and using this to inform personalised nutritional advice in patients with type 2 diabetes. The amount of professional experience ranged from 1 year to 22 years post-registration. The nutritionists provided nutritional advice in their capacity as independent healthcare professionals. Una Health developed a smartphone application for this study, which participants used to log record their activity. Participants were provided with a unique username and password which they used to log into the app. Once logged in, participants could record their meals, drinks, and exercise through a series of intuitive steps. For meals, subjects selected the meal type (breakfast, lunch, dinner, or snack) and then provided information about the meal in free text fields, such as the type of foods/ingredients eaten. If they wished, they could take a picture of their meals. Subjects were then asked if they were about to consume the meal, or if they had already consumed it, and if so, at what time the meal was consumed. For drinks, participants were simply asked to complete a free text field with the drink description, optionally provide a photo, and then confirm the time of consumption of the meal. For exercise, subjects could provide a free text description of the exercise, indicate the intensity of the exercise (low, moderate, or high), indicate the duration of the exercise, and confirm the time the exercise started. The record data for each participant was stored in a pseudonymised format on a secure S3 cloud storage bucket and linked with the corresponding CGM data. These data were also shared with the nutritionist assigned to each participant. The macronutrient composition of logged meals was calculated by the nutritionists using a widely used food database (DGExpert, Deutsche Gesellschaft für Ernährung e. V., Bonn, Germany). By combining CGM data (which was analysed according to published guidance [20]), the data collected in the smartphone app, meal macronutrient composition, and participants’ PPGRs to different macronutrient combinations were quantified. Nutritionists used these data to draw objective conclusions on an individual’s optimal diet in terms of better PPGRs, and how this would map onto the ingredients in the meals consumed by the individual.

All enrolled participants were provided with two sensors (Abbott FreeStyle Libre 2, Abbott GmbH, Wiesbaden, Germany). The study period was split into two phases, a pre-insight phase where participants wore the first sensor, and a post-insight phase where they wore the second one. The participants consented to the sharing of their CGM data at the end of each phase. Between the pre-insight and post-insight phase, nutritionists provided participants with their personalised nutritional recommendations based on an individual’s response to different ingredients in a form of a report that was shared electronically. Participants also discussed the findings of this report with their nutritionist in a 30 min video call. Following this, further CGM and meal logging data were collected during the post-insight phase. The post-insight phase was defined as being at least 6 days long to allow for participants to incorporate recommendations made by nutritionists in their diets.

### 2.3. Statistical Analysis of Baseline Participant Characteristics

As described in the Results section below, a total of 18 participants out of the original 42 enrolled were withdrawn or excluded from data analysis. In order to understand whether there were any differences in characteristics such as age, BMI, HbA1c, and presence of other diseases between included (24 participants) and excluded (18 participants), the Mann–Whitney U test for continuous data and the chi-squared test for categorical data were performed. Multiple linear regression analysis was performed to determine the associations between baseline participant characteristics and PPGR (median iAUC) outcomes. Both analyses were carried out using GraphPad Prism (Version 9.3.0 (345), San Diego, CA, USA) and the open-package statsmodels v0.13.2 in python. Significance was considered if *p* ≤ 0.05.

### 2.4. Statistical Analysis of Glycaemic Metrics

The glycaemic metrics analysed were the changes in median incremental area under the curve (iAUC) across meals, mean glucose, percentage time in hyperglycaemia (defined as glucose > 180 mg/dL), and AUC during the day (defined as from 06:00 to 23:59) normalised hourly, between pre-insight and post-insight phases. All participant data were stored in pseudonymised format on a secure S3 cloud storage bucket. The calculation of the glycaemic outcomes outlined above was carried out using a script written in Python 3.8, and the outputs were saved as csv files. Statistical analysis was carried out using GraphPad Prism (Version 9.3.0 (345), San Diego, CA, USA). For the analysis of statistical differences between the pre- and post-insight phases, the Wilcoxon signed-rank test (paired, two-tailed) was chosen. In dot line graphs, each dot represents one individual patient. Bar graphs or dot blots are presented as the median ± IQR. *p*-values are indicated as * *p* ≤ 0.05, ** *p* ≤ 0.01, *** *p* ≤ 0.001, or **** *p* ≤ 0.0001. A trend is considered with a *p*-value of *p* = 0.05 to 0.1.

### 2.5. Statistical Analysis of Patient-Reported Outcome Measures (PROMs)

The collection of data on patient-reported outcome measures was conducted via questionnaires distributed using Google Forms. Participants completed five questionnaires during the study, one at the beginning of the study and one at the end of each week on the study. The questions focused on chronic-disease-related well-being, including energy levels, anxiety around self-management, satiety with food, and difficulty in concentrating, and were derived from standard questionnaires in the field [21,22,23]. The response scale was a five-point response format, ranging from “disagree” to “agree”. Raw data were first transferred to sheets and statistically analysed using Prism (Version 9.3.0 (345), San Diego, CA, USA). Panel OLS regression with unit fixed effects in Python 3 was chosen for statistical analysis to model outcome measures for within-individuals. The standard error is clustered on each patient and the estimate parameter gives an indication of likelihood of change. *p*-values are indicated as * *p* ≤ 0.05, ** *p* ≤ 0.01, *** *p* ≤ 0.001, or **** *p* ≤ 0.0001.

### 2.6. Multivariate Regression Analysis of Macronutrients

Statistical analysis was performed using the open-source Pingouin package version 0.5.1. Pingouin is a statistical package written in Python 3 and based mostly on Pandas and NumPy. For 1486 meals from 24 patients, multivariate linear regression analysis was used to determine the relationships between the macronutrient composition of each meal calculated by nutritionists as described above, and the corresponding maximum glucose level reached within 3 h of meal ingestion. Two-sided *p* < 0.05 was considered statistically significant.

### 2.7. User Research Analysis

A total of 6 user interviews were conducted to identify patterns regarding users’ experiences over the course of the study. All interviews were held remotely via video call and followed a semi-structured interview structure. All interviews were audio-recorded, fully anonymised, and subsequently transcribed. Thematic analysis was conducted to investigate emerging themes related to the perceived effectiveness and impact of personalised nutritional advice and users’ attitude towards this. An initial code framework was developed after the analysis of the first two interviews and then iterated through four subsequent coding rounds. The results were then discussed with the whole study team to uncover coding gaps through the direct comparison of user quotes and different interpretations. The coding steps leading to a multidimensional codebook were carried out according to the guidelines of the SAGE Handbook of Qualitative Data Analysis [24].

## 3. Results

A total of 42 individuals were initially recruited between August and October 2021. Three participants withdrew from the study without giving a reason, while five other participants were withdrawn due to illness or hospitalisation over the course of the study. Ten further participants were excluded from final analysis due to insufficient glucose data being shared with the study team. No significant differences were identified between the included (*n* = 24) eligible participants and excluded/withdrawn (*n* = 18) participants. Specifically, no differences between the included and excluded participants in age (median ± IQR: 55 ± 15 years vs. 54 ± 14 years, *p* = 0.9696), in BMI (median ± IQR: 31.60 ± 8.85 kg/m^2^ vs. 33.60 ± 6.4 kg/m^2^, *p* = 0.3945), in HbA1c values (median ± IQR: 7.35 ± 1.8 % vs. 7.60 ± 1.2 %, *p* = 0.7616) and the presence of other diseases (*p* = 0.2568).

### 3.1. Baseline Participant Characteristics

A demographic analysis of the participants who completed the programme is presented in Table 2. The mean age of participants was 54 years (range 27–74). In total, 75.00% of participants were female and the mean weight was 95.78 ± 17.61 kg (mean ± SD), with the mean BMI of 31.87 ± 6.27 kg/m^2^ at start of the pilot. Moreover, 88.46% of participants were taking previously prescribed antidiabetic medication, and 50% were on more than one class of drug including metformin, GLP-1-agonist, SGLT-2-inhibitor, DPP-4, and alpha-glucosidase inhibitors. The mean HbA1c value was 7.92 ± 1.77%. The mean length of time since year of diagnosis was 6 ± 8 years, and the mean age of diagnosis was 46 ±12 years with the youngest person diagnosed at 17 years of age and the oldest 68 years of age. A total of 62.50% of patients were diagnosed with other conditions alongside type 2 diabetes including 33.33% with hypertension, 12.5% with autoimmune diseases, and 37.5% with other illnesses (including rheumatoid arthritis, liver cirrhosis, hypercholesterolaemia, hypertriglyceridaemia, and lymphoedema).

### 3.2. Individual Differences

**Figure 1:**
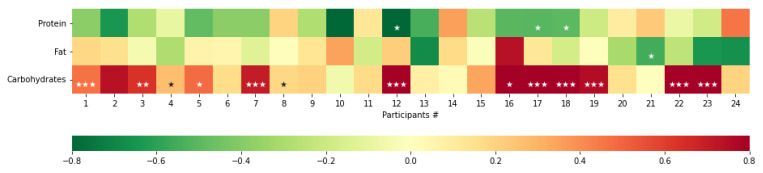
Identification of individual differences. Heatmap of regression coefficient from multivariate regression analysis on 24 patients, all of whom had at least 28 meals annotated with nutrition values. The number of stars (* *p* ≤ 0.05, ** *p* ≤ 0.01, *** *p* ≤ 0.001) represent significance of the linear relationship between nutrient and maximal glucose level (gmax). The value of the regression coefficient (colour) represents the strength of the positive/negative correlation between each nutrient and gmax. A red colour indicates that the increase in glucose level is more pronounced if the amount of the relevant macronutrient increases in the meal when controlling for the amount of other macronutrients.

As outlined in the Methods section, nutritionists calculated the absolute mass of carbohydrate, protein, fat, and fibre, as well as the calorie content of meals logged by participants. Multivariate linear regression analysis was used to determine the relationships between the macronutrient composition of each meal and the corresponding maximum glucose level within 3 h after the meal (gmax). Glycaemic responses to different macronutrients are not uniform across our cohort, with differing sensitivities to carbohydrate and differing effects of protein and fat content on gmax (Figure 1).

### 3.3. Glycaemic Metrics

**Figure 2:**
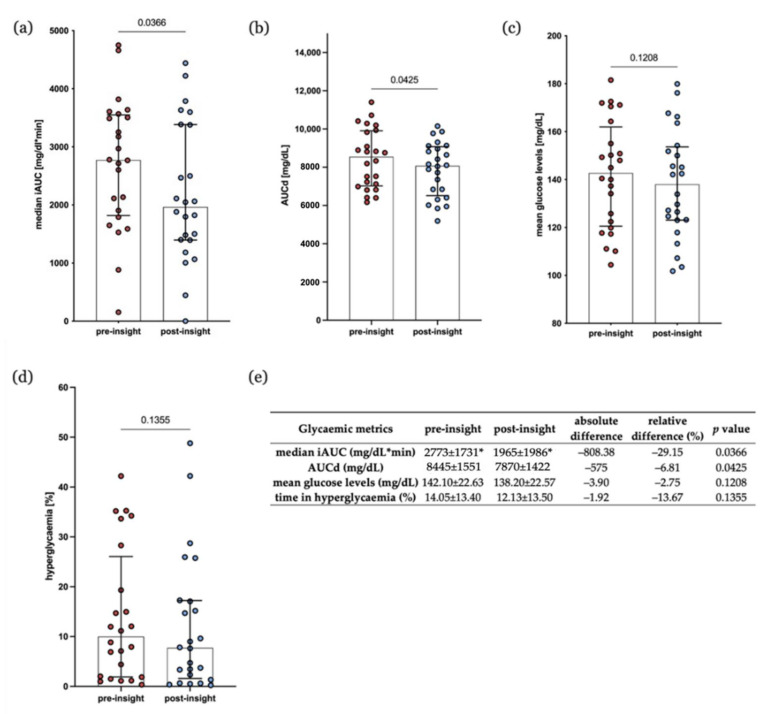
Glycaemic metrics pre- and post-insight phase. (**a**) The median incremental area under the curve (iAUC). (**b**) Daytime area under the curve (AUCd). (**c**) Mean glucose levels. (**d**) Percentage (%) of time spent in hyperglycaemia. (**e**) Summary statistics for (**a**–**d**) including relative difference (%), absolute difference, and *p* value. * For all metrics, mean ± SD is shown, except for median iAUC, for which median ± IQR is shown. (**a**–**d**) Each dot represents one individual participant. Wilcoxon matched-pair signed ranked test was chosen to test for significance; error bars show median ± IQR; *n* = 24.

To examine the effects of personalised nutritional analysis on glycaemic metrics, the mean glucose, time in hyperglycaemia, the incremental area under the curve (iAUC) after each meal, and the daytime area under the curve normalised hourly (AUCd) were calculated. The results in the time period before participants were provided with their report were compared to those after the participants received their report (Figure 2). Figure 2e provides a summary of the results. Table S1 in Supplementary Information provides the results for all participants whose data were analysed, comparing pre- and post-insight phases.

The iAUC quantifies the post-prandial response to meals. For each time period, the median iAUC across all meals logged in that period was calculated, and the medians were compared for all participants between time periods (Figure 2a). The median of the median iAUCs across all participants decreases by 29.15% from the pre- to post-insight phase (median ± IQR: 2773 ± 1731 vs. 1965 ± 1986 *p* = 0.0366). The AUCd also decreases to a statistically significant degree between study phases by 6.81% (median ± IQR: 8547 ± 2887 vs. 8074 ± 2560, *p* = 0.0425, (Figure 2b). The mean glucose levels and time in hyperglycaemia did not differ significantly between pre- and post-insight phases (Figure 2c,d)). These results suggest that the provision of personalised nutritional analysis to patients with type 2 diabetes improves post-prandial glycaemic response and overall daytime glucose exposure.

Multiple linear regression analysis was performed to investigate the associations between major baseline characteristics (age, BMI, HbA1c, and the presence of other diseases) and the effects on PPGR outcomes. The analysis indicates that none of these characteristics have an effect on median iAUC in the pre-insight phase, post-insight phase, or on the absolute difference (changes) after both phases, as depicted in Table 3.

### 3.4. Participant-Reported Outcome Measures

**Figure 3:**
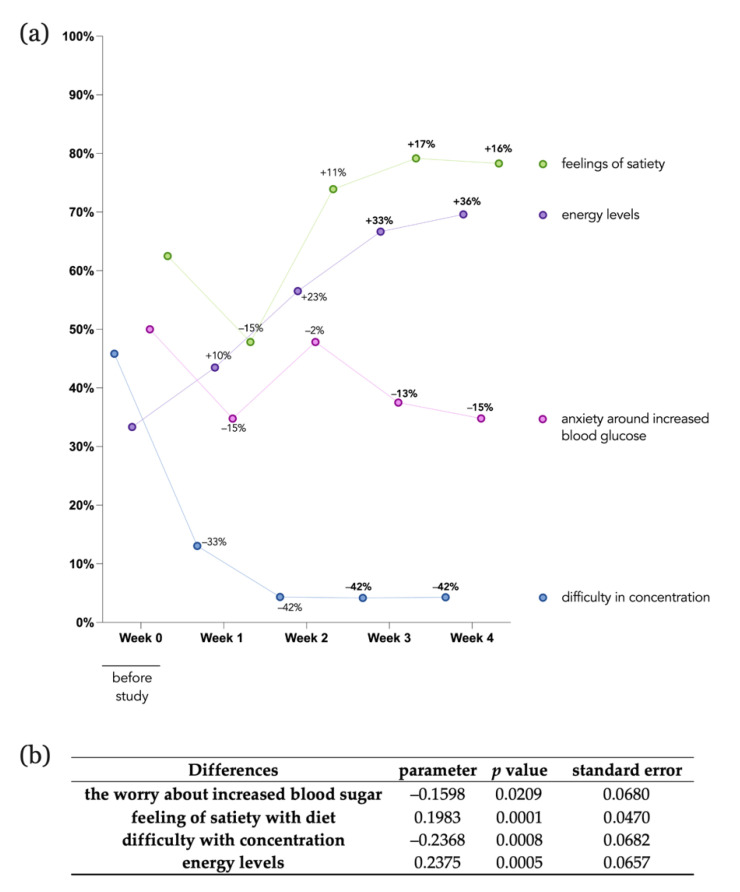
Participant-reported outcome measures. Self-reported energy levels, anxiety around increased blood glucose, feelings of satiety, and difficulty in concentration were measured weekly over the course of the programme. (**a**) Percentage (%) of participants agreeing or strongly agreeing with the statement are shown. Percentages (%) depicted directly in the line-dot graph describe the relative weekly difference compared to baseline questionnaire. (**b**) Panel OLS fixed-effect model regression analysis outcomes including estimated parameter, *p*-values, and standard error for each question is shown. Time was used as the independent variable (Y) and the question as the dependent variable (X) for the within-individual effects, and the standard error was clustered for one person. Data were tested for normal distribution.

To assess participants’ well-being over the course of the study, five participant-reported outcome measures (PROMs) surveys were conducted [21,22,23,25]. The first baseline questionnaire was filled out on the first day of the programme, and the subsequent four surveys were filled out at the end of each week. Figure 3 provides the results obtained from the surveys to measure the extent to which the energy levels, worrying about increased blood sugar, satiety from diet, and difficulty in concentrating changed over the course of the programme. Changes in self-reported ability to concentrate were notable one week after the start of the programme, with the number agreeing or strongly agreeing that they experienced such difficulties in the last week declining by −33% compared to the baseline questionnaire; this number decreased further by −42% in week 2 and was maintained into the last week. When patients were asked to rate their agreement to the statement “Over the last week, I felt like I had a lot of energy”, only 33% agreed or strongly agreed in the baseline questionnaire, but this increased to almost 70% by the end of the programme. Half of the participants reported that they were worried about their glucose levels being too high at the beginning of the programme, but the proportion expressing this view decreased by 15% by the end of the programme. A total of 63% of participants agreed or strongly agreed that they felt contented and sated with their diet before the start of the programme, and the number of patients agreeing to this question increased weekly after week 2 (Figure 3).

Fixed-effects model regression analysis was performed in order to analyse individual-specific PROMs dependent on the scaling (1–5, disagree–agree) over time. The table depicted in Figure 3b shows the estimated parameter, *p* value, and standard error calculated for each question. Individuals worried significantly (*p* value = 0.00209) less about their blood sugar by −0.1598 points weekly. The feeling of satiety with diet improved significantly (*p* value = 0.0001) by 0.1983 points weekly. Moreover, a significant reduction (*p* value = 0.0008) in difficulty to concentrate weekly by −0.2368 points was observed. Lastly, the fixed-effects model estimated a significant increase in energy level by 0.2375 points weekly. Overall, the differential analysis and fixed-effects model regression analysis indicate substantial improvement in energy levels, a reduction in worrying about increased blood glucose, improvement in feelings of satiety, and a reduction in difficulty in concentration over the course of the study period.

### 3.5. User Research

Thematic analysis was performed to gain understanding and identify user patterns around three areas: overall user engagement, the effectiveness of the programme, and users’ priorities and needs. The result of the thematic analysis can be explained through a codebook, which summarises the main themes identified. All codes are shown with exemplary quotes that elaborate users’ experience with the programme. Notably, three overarching themes were identified: the perceived impact of knowledge gained by CGM, acquiring knowledge of the individual reactions to different foods, and pain points including self-management barriers. The respective subthemes are listed in Table 4 below.

## 4. Discussion

Previous research has demonstrated strong and consistent inter-individual differences in post-prandial glycaemic responses to meals [7,8]. Moreover, controlled lifestyle-interventional trials for type 2 diabetes patients have demonstrated the limited efficacy of general nutritional advice in improving glycaemic control [7,27,28,29,30]. These findings suggest that nutritional recommendations need to be personalised. Our observational study set out to evaluate the effect of CGM-informed personalised nutritional interventions on glycaemic metrics and self-reported well-being, as well as to better understand participant priorities and needs, with a view to determining the value of developing a digital health product that delivers personalised nutritional intervention. Importantly, our focus was on free-living individuals who did not eat solely standardised meals but their normal diet. Diet is strongly influenced by sociocultural factors [31,32], and to our knowledge, this is the first study focusing on free-living people with type 2 diabetes in Germany with their individual diets.

In our study, individual participants reacted differently to various macronutrients in terms of their post-prandial glycaemic responses (PPGRs). This is in line with the work from Berry and colleagues, who observed considerable inter-individual differences in PPGRs to standardised meals [7]. Our analysis provides insights into free-living people eating in non-experimental conditions following their normal dietary habits. We show that even in a small group with a non-standardised diet, there are clear intra-individual differences in the reaction to macronutrients, highlighting again the basis for personalised nutritional advice.

Since this study was observational and was not powered to detect a particular change in glycaemic metrics, it is unsurprising that reductions in mean glucose levels and time spent in hyperglycaemia were not statistically significant. Nevertheless, the observation of statistically significant improvements in median iAUC and AUCd suggest that providing patients with their personalised assessment of PPGRs, including how they react to specific macronutrient combinations, and with nutritional advice based on this, improves their glycaemic control.

Our investigation also revealed significant improvements in participants’ self-reported outcomes regarding their energy levels, anxiety around increased blood glucose, feelings of satiety, and difficulty in concentration, suggesting that providing participants with personalised nutritional analysis improves their well-being. This study was not designed to determine the impact of this improved well-being on glycaemic control, but this merits further research, particularly given the link between anxiety and poor control [33,34].

User research interviews were held to examine the role of personalised support delivered via a digital health application on the participants’ understanding and knowledge of type 2 diabetes and motivation to adhere to lifestyle modifications. Such user research is essential in the early stages of successful digital product development [35] and has been identified by the WHO as a specific focus area for digital health research [36]. To our knowledge, this study is the first to explore user requirements from such an intervention. Our data demonstrate that access to personalised support in combination with CGM delivered through a digital application improves users’ awareness, knowledge, and motivation to sustain nutritional and lifestyle changes. Our findings corroborate the ideas of Fu and colleagues, who suggested that both personalisation and instant feedback, including motivational self-management support, educational information about individual glucose variabilities, and personalised reports, would lead to improvements in adherence [37].

As this study was observational and conducted with the aim of assessing the value of developing a digital health product to support personalised nutritional intervention for patients with type 2 diabetes in Germany who are not on insulin, there are a number of limitations with respect to the interpretation of the results and generalising them to the management of type 2 diabetes more widely. Firstly, participants were recruited using social media, which may create bias for increased engagement with the management of their diabetes and increased digital literacy. Future studies will ensure broader recruitment to account for different levels of digital literacy and engagement in the wider type 2 diabetes population.

Another limitation of this observational study was that the ‘intervention’ was not standardised, with the five nutritionists relying on their professional judgement in the provision of nutritional recommendations. Moreover, participants took part in the study for approximately four weeks, and there was no follow-up to ascertain whether improvements in glycaemia or well-being persisted long-term. As such, future studies will recruit sufficient participants to be adequately powered to detect changes in glycaemic metrics, will be designed to compare the effect of a personalised nutritional intervention to standard of care and to CGM alone, will run for over three months to ensure that changes in HbA1c can be measured [38], and will include a follow-up phase to assess the long-term impact of the intervention.

## 5. Conclusions

This study provides important insights into the desirability of an intervention based on personalised nutritional advice for a group of patients with type 2 diabetes, as well as the potential efficacy of such an intervention on glycaemic control, self-reported well-being, and motivation to maintain diabetes self-management. A major strength of this study is that it involved the observation of free-living individuals who were eating their normal diet, rather than standardised meals. These findings suggest that developing a digital health product for patients with type 2 diabetes that delivers personalised metabolic insight based on nutritional advice could guide users to make healthier decisions and implement sustainable lifestyle modifications.

## Figures and Tables

**Table 1:** Eligibility criteria.

Inclusion	Exclusion
Diagnosis of type 2 diabetes	Pregnancy or breastfeeding
Age ≥ 18 years	Treated with insulin, sulphonylureas, glinides
Own and are able to operate a smartphone	Unable to consent to involvement in research
	Episodes of symptomatic hypoglycaemia in the last 3 months

**Table 2:** Demographic characteristics of eligible participants.

Demographics	Count *n* = 24	Descriptive Statistics *n* = 24
Age		
<30 to 40	3 (12.50%)	
41 to 50	7 (29.17%)	
51 to 60	7 (29.17%)	
61 to 70	5 (20.83%)	
71 to 80	2 (8.33%)	
Mean ± SD		54 ± 11.18 years
Maximum		74 years
Minimum		27 years
**Gender**		
Female	18 (75.00%)	
Male	6 (25.00%)	
**HbA1c**		
<6.5%	2 (8.33%)	
6.5% to 6.9%	6 (25.00%)	
7% to 7.9%	6 (25.00%)	
8% to 8.9%	5 (20.83%)	
9% to 10%	3 (12.50%)	
>10%	2 (8.33%)	
Mean ± SD		7.92 ± 1.77%
Maximum		13.40%
Minimum		5.30%
**Weight**		
Mean ± SD		95.78 ± 17.61 kg
Maximum		125.40 kg
Minimum		61 kg
**BMI ^1^ (kg/m^2^)**		
Mean ± SD		31.87 ± 6.27 kg/m^2^
Maximum		44.43 kg/m^2^
Minimum		21.36 kg/m^2^
Median		31.59 kg/m^2^
**Year of diagnosis**		
<2000	1 (4.17%)	
2000 to 2009	3 (12.50%)	
2010 to 2019	12 (50.00%)	
>2020	8 (33.33%)	
Mean ± SD		2015 ± 8 years
Maximum		2021
Minimum		1986
**Age at diagnosis**		
<31	3 (12.50%)	
31 to 40	1 (4.17%)	
41 to 50	11 (45.83%)	
51 to 60	6 (25.00%)	
61 to 70	3 (12.50%)	
Mean ± SD		47 ± 11.81 years
Maximum		68 years
Minimum		17 years
**Antidiabetes medication**		
No	3 (11.54%)	
Yes	21 (88.46%)	
1 type of AM	9 (37.50%)	
>1 type of AM	12 (50.00%)	
metformin	19 (79.17%)	
GLP-1-agonist ^1^	4 (16.67%)	
SGLT-2-inhibitor ^1^	6 (25.00%)	
DPP-4 ^1^	7 (29.17%)	
Alpha-glucosidase inhibitors	1 (4.17%)	
**Other conditions**		
No	9 (37.50%)	
Yes	15 (62.50%)	
Hypertension	8 (33.33%)	
Autoimmune disease	3 (12.50%)	
Other illness ^1^	9 (37.50%)	

^1^ AM, antidiabetes medication; BMI, body mass index; DPP-4, dipeptidylpeptidase4; GLP-1, glucagon-like peptide-1 receptor; HbA1c, haemoglobin A1c; SD, standard deviation; SGLT-2, sodium glucose cotransporter-2; other illnesses included rheumatoid arthritis, liver cirrhosis, hypercholesterolaemia, hypertriglyceridaemia, and lymphoedema.

**Table 3:** Multiple linear regression analysis of baseline characteristics and glycaemic metrics.

Major Baseline Characteristics	Regression Coefficient (Beta)	Standard Error of the Estimate	*p* Value
Pre-insight median iAUC			
Age	5.566	0.836	0.836
HbA1c	118.845	0.437	0.437
BMI	−17.832	0.697	0.697
Other diseases	68.698	0.903	0.903
**Post-insight median iAUC**			
Age	1.822	0.950	0.950
HbA1c	−36.546	0.824	0.824
BMI	6.180	0.901	0.901
Other diseases	−174.313	0.775	0.775
**Absolute difference median iAUC**			
Age	−3.744	0.884	0.884
HbA1c	−155.391	0.289	0.289
BMI	24.012	0.583	0.583
Other diseases	−243.010	0.651	0.651

**Table 4:** Coding frame and example codes as a result of the thematic analysis conducted on 6 user interviews.

Themes	User Quotes
The perceived impact of knowledge gained by CGM	
Altered meal planning behaviour	The bad meals, so to say, don’t make it on my grocery list. (U4)
Recycling of good ingredients for other meals	I looked at what was positive and then I also looked at which building blocks I could use or could use later and perhaps modify them a little and then make another meal out of it that would have the same positive effect. (U3)
Decreasing proportions of less ideal ingredients	Or take one tablespoon or two tablespoons less oatmeal. Well, you’ll still be full with the nuts you add and everything. But take a little less of it. And I found that really very, very pleasant to be able to observe over these four weeks that there had been a clear improvement. (U5)
Reduction in hidden sugars	But I eat much, much more consciously. I always try to include nuts and things like that, things that have value, and I really refrain from anything that basically also contains hidden sugars and things like that. I stick to that consistently. (U6)
Increased motivation	I was somehow relieved. I didn’t do everything wrong. Before I really thought I was doing everything wrong when it was so high. And yes, I’m highly motivated, totally motivated. (U2)
**Acquiring knowledge of the individual reactions to different foods**	
Meal analysis of meal components	But you suddenly notice it, or at least I do, much more consciously, because otherwise you don’t dissect and pick apart your meal like that. (U5)
Personalised meal analysis and recommendations	With so many programmes, I would say, or dietary change programmes, a lot of things are given to you and you have to integrate things that you might not like so much. They also include foods that you don’t use in your routine. And I actually found it very, very good that I got information about the value of my diet tailored to my meal plan, to my family’s daily routine. (U1)
Combination of CGM curve and meal logging	But I can also link that [CGM values] to the meal. That I can then see okay, I ate this and that and afterwards it rises and falls again in that specific period of time. This is also possible in the morning or at lunchtime with every meal, so that it is easier to follow up than if I were to take a blood sample. (U3)
**Pain points and self-management barriers**	
Weight reduction	If I want to lose weight, I also have to pay a bit of attention to how these macronutrients are and what influence they have. So in this case, not only for blood sugar, but also overall. (U3)
Lifestyle barriers	Testing the meals, that is actually something that I find very interesting. And actually, I would have liked to do all of them, but that is quite difficult for me in my everyday life. Because I can’t eat or drink anything else for two hours before and two hours after the meal. (U1) *

* The user referred to the time span of 2 h required between two meals to calculate reliable meal analysis results [26].

## Data Availability

The data presented in this study are available on request from the corresponding author, provided that they will be used for genuine scientific purposes and that these purposes will not compromise their utility in future product research and development. The data are not publicly available due to ongoing analysis for product research and development.

## Supplementary Materials

The following supporting information can be downloaded at: https://www.mdpi.com/article/10.3390/nu14102123/s1, Table S1: Glycaemic metric data for all participants whose data were analysed in pre- and post-insight phases.
