# Supplementary material for: Personalised Nutritional Recommendations Based on Individual Post-Prandial Glycaemic Responses Improve Glycaemic Metrics and PROMs in Patients with Type 2 Diabetes: A Real-World Assessment
Source: Nutrients. 2022 May 19;14(10):2123. doi: 10.3390/nu14102123 (PMC9145975; doi:10.3390/nu14102123)
Supplement: Supplementary file 1 [file nutrients-14-02123-s001.zip › nutrients-1714040-supplementary.pdf]

Supplementary Materials:

Table S1. Glycaemic metric data for all participants whose data was analysed in pre- and post-insight phases.

	Pre- insight				Post-insight			
**Participant**	**median iAUC (mg/dl*min)**	**AUCd (mg/dl)**	**Time in hyperglycaemia (%)**	**MG (mg/dl)**	**median iAUC (mg/dl*min)**	**AUCd (mg/dl)**	**Time in hyperglycaemia (%)**	**MG (mg/dl)**
01	4749.25	7526.10	4.40	119.88	4440.00	5948.71	7.64	126.50
02	2715.00	11403.72	42.21	181.55	1395.00	9861.42	28.72	163.59
03	1528.25	6397.21	0.35	104.41	2498.50	9767.53	2.35	113.24
04	2113.50	6167.63	6.90	147.99	1005.00	5192.26	3.36	142.09
05	3505.50	8811.43	11.97	140.47	2062.50	8419.31	4.73	133.93
06	1790.00	8191.93	7.92	140.00	1797.00	5845.77	3.73	124.67
07	3606.00	8333.12	7.11	134.10	1822.50	6308.64	0.23	107.21
08	1649.25	7123.66	2.02	117.70	2047.50	8101.50	0.62	117.89
09	2767.50	9934.40	14.70	150.86	1882.25	8820.92	9.00	145.14
10	2779.00	6809.37	1.13	111.08	0.00	8045.65	3.47	123.23
11	2135.00	9080.03	8.85	137.31	3379.00	7891.32	7.83	129.65
12	154.50	6411.31	28.30	164.30	1183.50	8159.53	42.23	176.16
13	883.00	9831.50	19.32	155.05	1480.75	9136.55	17.27	154.21
14	3818.75	10412.99	35.24	170.50	3633.00	8647.43	48.79	179.92
15	2604.00	8833.03	14.97	149.34	1401.00	7354.59	17.08	145.56
16	3489.30	7230.66	1.87	117.30	1502.10	6019.39	0.35	101.78
17	4659.75	8760.13	11.14	144.98	3598.65	10152.8[illegible]	14.71	150.03
18	3252.60	6821.58	1.51	110.14	3384.90	6407.67	1.34	103.50
19	1588.50	6988.26	0.99	122.43	1066.50	6846.76	0.66	127.17
20	3565.35	7510.57	1.16	125.80	3786.75	6843.51	0.52	123.04
21	3637.00	10717.27	34.22	172.58	2111.00	9307.64	25.74	167.70
22	3175.65	10286.49	35.20	171.12	2467.80	8961.15	15.18	151.81
23	2970.00	8894.50	12.06	150.20	4222.50	7723.11	9.63	142.44
24	1904.50	10197.06	33.65	171.98	445.00	9116.25	25.95	166.27
